# Predictive distributions were developed for the extent of heterogeneity in meta-analyses of continuous outcome data

**DOI:** 10.1016/j.jclinepi.2014.08.012

**Published:** 2015-01

**Authors:** Kirsty M. Rhodes, Rebecca M. Turner, Julian P.T. Higgins

**Affiliations:** a MRC Biostatistics Unit, Cambridge Institute of Public Health, Forvie Site, Robinson Way, Cambridge Biomedical Campus, Cambridge, CB2 0SR, UK; b School of Social and Community Medicine, University of Bristol, Canynge Hall, 39 Whatley Road, Bristol, BS8 2PS, UK; c Centre for Reviews and Dissemination, A/B Block, Alcuin College, University of York, York, YO10 5DD, UK

**Keywords:** Meta-analysis, Heterogeneity, Intervention studies, Bayesian analysis, Continuous data, Standardized mean difference

## Abstract

**Objectives:**

Estimation of between-study heterogeneity is problematic in small meta-analyses. Bayesian meta-analysis is beneficial because it allows incorporation of external evidence on heterogeneity. To facilitate this, we provide empirical evidence on the likely heterogeneity between studies in meta-analyses relating to specific research settings.

**Study Design and Setting:**

Our analyses included 6,492 continuous-outcome meta-analyses within the Cochrane Database of Systematic Reviews. We investigated the influence of meta-analysis settings on heterogeneity by modeling study data from all meta-analyses on the standardized mean difference scale. Meta-analysis setting was described according to outcome type, intervention comparison type, and medical area. Predictive distributions for between-study variance expected in future meta-analyses were obtained, which can be used directly as informative priors.

**Results:**

Among outcome types, heterogeneity was found to be lowest in meta-analyses of obstetric outcomes. Among intervention comparison types, heterogeneity was lowest in meta-analyses comparing two pharmacologic interventions. Predictive distributions are reported for different settings. In two example meta-analyses, incorporating external evidence led to a more precise heterogeneity estimate.

**Conclusion:**

Heterogeneity was influenced by meta-analysis characteristics. Informative priors for between-study variance were derived for each specific setting. Our analyses thus assist the incorporation of realistic prior information into meta-analyses including few studies.

**What is new?**

**Key findings**
• This article represents a very large empirical study of continuous-outcome meta-analyses, showing that meta-analysis characteristics strongly influence the extent of heterogeneity.
• Predictive distributions have been obtained for the expected between-study variance in future meta-analyses, and these differ substantially across settings defined by outcome type, type of intervention comparison, and medical area.

**What this adds to what was known?**
• When a meta-analysis includes a small number of studies, estimation of the between-study variance is difficult. The existing literature on heterogeneity in meta-analyses of continuous outcomes is sparse, and so little is known as to what forms a realistic prior distribution for the between-study variance. This article proposes a new set of informative prior distributions for use in specific research areas.

**What is the implication and what should change now?**
• We have demonstrated how an informative prior for heterogeneity can be used in a future meta-analysis. In each of two illustrative examples, incorporation of external information led to more precise estimates for the between-study variance.
• In view of the strong associations between meta-analysis characteristics and the extent of heterogeneity observed in our data set, the use of an empirically derived informative prior for heterogeneity in future meta-analyses would be perfectly reasonable.

## 1 Introduction

Policy decision makers are becoming increasingly reliant on the findings from systematic reviews [1]. Within systematic reviews are meta-analyses that combine results from similar studies to synthesize available evidence in a specific research area. Variation among the results of included studies, known as heterogeneity, is inevitable. The studies have likely been conducted using different methods, at various locations, and by different teams. Statistical heterogeneity occurs when the variation between study results is greater than that expected by chance. Several possible approaches are available to deal with heterogeneity: we can ignore it, investigate it, or we may decide not to perform a meta-analysis at all. Alternatively, we can allow for heterogeneity in a random-effects meta-analysis, estimating the summary effect and the between-study variance [2].

In many meta-analyses, there are few studies available to include, perhaps because the disease is rare or the treatment under assessment is new. Of 22,453 meta-analyses from the Cochrane Database of Systematic Reviews (CDSR), containing at least two studies, just under 75% contained five or fewer studies [3]. When there are only a small number of studies included in a meta-analysis, estimation of the between-study variance is difficult. In a conventional random-effects meta-analysis, the uncertainty in the between-study variance is not accounted for [2]. However, within a Bayesian framework, we can allow for all sources of uncertainty and incorporate external evidence on heterogeneity. To perform a Bayesian random-effects meta-analysis, prior distributions need to be specified for unknown parameters. It has been recommended that a realistic prior distribution should be used for the between-study variance [4], [5], [6].

To facilitate Bayesian meta-analysis with an informative prior for the between-study variance, we provide empirical evidence on the likely extent of heterogeneity in meta-analyses of particular settings, defined by outcome type, types of interventions evaluated, and medical area. Study data from the binary outcome meta-analyses in the CDSR have already been analyzed by Turner et al. [5]. Turner et al. summarized a set of informative prior distributions for the between-study variance *τ*^2^ for use in future binary outcome meta-analyses on the log odds ratio scale.

Here, we analyze data from a large collection of published continuous-outcome meta-analyses and investigate the influence of meta-analysis characteristics on between-study heterogeneity. We provide predictive distributions for the extent of heterogeneity expected in future continuous-outcome meta-analyses in particular settings. These distributions can be used in new meta-analyses as “off-the-shelf” informative prior distributions for the between-study variance [4], [7].

## 2 Methods

### 2.1 Data description

CDSR is a rich resource of systematic reviews in areas of health care. These reviews have been prepared by the Cochrane Collaboration, with the objective to make the most up-to-date and reliable evidence conveniently available to health care consumers, professionals, and providers [3]. In this research, data from the CDSR (issue 1, 2008) were provided by the Nordic Cochrane Centre.

Cochrane reviews typically include multiple meta-analyses, which correspond to the comparisons of different pairs of interventions or the assessment of different outcomes within the same research area. For example, a review examining antibiotics could report separate meta-analyses comparing each of several antibiotics against a placebo, with respect to both infection severity and adverse effects. Meta-analyses were included in our analyses if they consisted of data from at least two studies. In some reviews, results from studies eligible for a meta-analysis were available, but no pooled results were published in the Cochrane review. Such data were regarded in the same way as meta-analyses to maximize the amount of information available. The review authors may have decided not to perform a meta-analysis based on the degree of heterogeneity between studies [3].

Reviews sometimes present results for several subgroup analyses within meta-analyses. Because we are interested in the overall between-study heterogeneity in a meta-analysis, study results were combined across subgroups. In some reviews, the subgroups presented within a meta-analysis were not mutually exclusive; therefore, we checked for study duplications and used data for only the first occurrence of each study in each meta-analysis [3].

All meta-analyses in the original CDSR database have been classified according to the type of outcome, types of interventions involved in the pairwise comparison, and medical specialty, as described in an earlier article [3]. In previous work conducted on binary outcome meta-analyses, Turner et al. [5] classified types of outcome according to three categories (objective, semiobjective, and subjective). When grouping outcomes for the analyses of continuous data, we decided to use narrower outcome groupings because there were no continuous outcomes we judged to be objective and fewer outcome categories in total.

For each study measured as a continuous outcome, we have study data consisting of means and standard deviations, together with the number of participants in each intervention group. All meta-analyses have been categorized according to whether the meta-analysis was originally published on the mean difference (MD) or standardized mean difference (SMD) scale.

### 2.2 Statistical analysis

We used hierarchical models to analyze study data from each meta-analysis in the data set, while investigating the influence of meta-analysis characteristics on the extent of between-study heterogeneity. Within each meta-analysis, a random-effects model with normal within-study likelihoods was fitted to continuous outcome data from each study, on the SMD scale. A definition of the SMD is provided in the Appendix at www.jclinepi.com (Section A.1.1).

Many meta-analyses in the data set have been published on the MD scale. Nonetheless, we analyzed all study data using the SMD scale, and we compared the distribution of heterogeneity among observed SMDs for meta-analyses originally analyzed on the SMD scale and meta-analyses originally analyzed on the MD scale. Our analyses initially investigated the distributional form of the between-study heterogeneity variance *τ*^2^, without accounting for meta-analysis characteristics as covariates. We contemplated several distributions for *τ*^2^ and took forward three distributions into later analyses, adjusting for covariates, based on assessment of goodness of fit. Turner et al. [5] and Pullenayegum [6] fitted a normal distribution to log-transformed values of underlying between-study heterogeneity *τ*^2^ in binary outcome meta-analyses. We contemplated a log-normal distribution for *τ*^2^ in continuous-outcome meta-analyses. Other candidate distributions included the heavier tailed log-*t* distribution with five degrees of freedom, and also an inverse-gamma distribution, as a conjugate prior for the variance of a normal distribution. Model selection based on the deviance information criterion (DIC) [8] led to the choice of the log-*t* model for *τ*^2^.

Across meta-analyses, a hierarchical regression model was fitted to log-transformed values of underlying between-study heterogeneity, assuming a *t* distribution with five degrees of freedom for residual variation. As covariates in our regression models, we included indicators for outcome type, type of intervention comparison, and medical area. Within pairwise comparisons, heterogeneity was assumed to vary across meta-analyses, with separate variances for the different outcome types. Heterogeneity was also assumed to vary across pairwise comparisons, with separate variances for each type of intervention comparison. The mathematical form of the model is given in the Appendix at www.jclinepi.com (Section A.1.2).

All models were fitted using Markov chain Monte Carlo (MCMC) within the WinBUGS [9] software (MRC Biostatistics Unit, Cambridge), and results were based on 50,000 iterations after a burn-in period of 10,000 iterations. This was sufficient to achieve convergence. Convergence diagnostics were run on the 50,000 iterations after burn-in. We monitored convergence using the Brooks–Gelman–Rubin statistic [10], as implemented in WinBUGS. For each MCMC, convergence was checked graphically via trace plots and autocorrelation plots. Vague normal (0,10) priors were declared for all regression coefficients, as recommended by Spiegelhalter et al. [11]. We tried a range of plausible vague prior distributions for the scale parameters of the random effects. An inverse-gamma (0.1,0.1) distribution was found to provide the best overall performance and was therefore assigned to each scale parameter in all analyses.

For each setting defined by outcome type, type of intervention comparison, and medical area, we obtained a predictive distribution for the between-study heterogeneity variance $\tau_{new}^{2}$ expected in a future meta-analysis in that setting, within the full Bayesian model. The algebraic form of the predictive distribution for $\tau_{new}^{2}$ is provided in the Appendix at www.jclinepi.com (Section A.1.2). A log-*t* distribution was fitted to each predictive distribution, using posterior quantities for log ($\tau_{new}^{2}$). This process provided parametric distributions approximating the predictive distributions under the full Bayesian model. These distributions are easily summarized and can serve as prior distributions for *τ*^2^ in future meta-analyses [4], [7]. In earlier work carried out on binary outcome meta-analyses, outcome types were categorized into three broad groups. Here, we grouped continuous-outcome meta-analyses into narrower categories by outcome type, providing an extensive library of informative priors for heterogeneity.

## 3 Results

### 3.1 Descriptive analyses

The data set includes 6,672 continuous-outcome meta-analyses, containing data from 29,902 studies. Of these meta-analyses, 79% (5,280 meta-analyses) were originally performed on the MD scale, and 21% (1,392 meta-analyses) were originally performed on the SMD scale. Seven hundred twenty-eight studies (2.4%) have missing standard deviations and are therefore removed from our statistical analysis. Table 1 lists the structure of the data set used for our analyses.

**Table 1:** Structure of the data set

	*N*	Min	Median	Max	IQR
No. of comparisons per review	1,138 reviews	1	1	22	1–2
No. of meta-analyses per comparison	1,949 comparisons	1	2	31	1–4
No. of studies per meta-analysis	6,492^a^ meta-analyses	2	3	98	2–5
Sample size	28,981^b^ studies	4	61	18,850	33–140

*Abbreviations*: Min, minimum; Max, maximum; IQR, interquartile range.

a We excluded 28 meta-analyses in which the outcome type did not fit into any of our predefined categories and was classified as “other.”

b We removed 728 studies with missing standard deviations of mean responses.

Twenty-eight meta-analyses (0.4%) were excluded from our analyses in which the outcome type did not fit into any of our predefined categories and was classified as “other.” Frequencies of outcome types, types of intervention comparison, and medical areas among the remaining 6,492 meta-analyses in our data set are given in Table 2.

**Table 2:** Ratios of between-study variances representing comparisons of heterogeneity among different types of meta-analyses, according to outcome, intervention comparison, and medical specialty

Meta-analysis type	No. of meta-analyses (%)	Ratio of *τ*^2^ (95% CI)
Outcome type		
General health-related outcomes^a^	1,300 (20)	1 (Reference)
Obstetric outcomes	165 (3)	0.39 (0.21, 0.69)
Resource use and hospital stay/process	456 (7)	1.78 (1.22, 2.52)
Internal and external structure-related outcomes	175 (3)	2.13 (1.05, 3.87)
Signs/symptoms reflecting continuation/end of condition and infection/onset of new acute/chronic disease	2,490 (38)	1.22 (0.93, 1.56)
Mental health outcomes	535 (8)	1.22 (0.84, 1.70)
Biological markers	1,053 (16)	0.84 (0.60, 1.15)
Various subjectively measured outcomes^b^	318 (5)	1.51 (1.05, 2.17)
Intervention comparison type		
Nonpharmacologic^c^ vs. any intervention	2,904 (45)	1 (Reference)
Pharmacologic vs. placebo/control	2,384 (37)	0.88 (0.63, 1.21)
Pharmacologic vs. pharmacologic	1,204 (19)	0.68 (0.42, 0.98)
Medical specialty		
Cardiovascular	475 (7)	1 (Reference)
Cancer	24 (0.4)	10.4 (2.50, 45.8)
Central nervous system/musculoskeletal	712 (11)	0.47 (0.29, 0.72)
Digestive system	1,144 (18)	1.06 (0.75, 1.57)
Infectious diseases	143 (2)	0.56 (0.27, 1.16)
Mental health and behavioral conditions	886 (14)	0.42 (0.28, 0.60)
Obstetrics and gynecology	671 (10)	1.14 (0.74, 1.76)
Pathologic conditions	254 (4)	0.87 (0.49, 1.54)
Respiratory diseases	1,345 (21)	0.12 (0.07, 0.18)
Urogenital	341 (5)	1.04 (0.63, 1.70)
Other	497 (8)	0.73 (0.43, 1.16)

*Abbreviations*: CI, credible interval.

a General health–related outcomes include general physical health, adverse events, pain, and quality of life/functioning.

b Various subjectively measured outcomes include consumption, satisfaction with care, composite end point (including at most one mortality/morbidity end point), and surgical or device-related success/failure.

c Nonpharmacologic interventions include interventions classified as medical devices, surgical, complex, resources and infrastructure, behavioral, psychological, physical, complementary, educational, radiotherapy, vaccines, cellular and gene, and screening.

In approximately 40% of meta-analyses analyzed originally on the SMD scale, the method-of-moments estimate for *τ*^2^ on this scale was negative and hence set to zero. Nonzero estimates for *τ*^2^ have a median of 0.10 and 95% range of 0.002–2.30. Among the meta-analyses analyzed originally on the raw MD scale but reanalyzed on the SMD scale, 43% of method of moment–based estimates for *τ*^2^ were negative and hence set to zero. Nonzero estimates for *τ*^2^ have a comparable median and 2.5% quantile to the meta-analyses analyzed originally on the SMD scale, a median of 0.11 and 95% range of 0.002–4.38. Histograms representing the empirical distributions of nonzero estimates for *τ*^2^ on the log scale are provided in the Appendix at www.jclinepi.com (Section A.2). The distributions based on analyses of MDs and SMDs are broadly similar, and in the remainder of the article, we use the complete data set, analyzed throughout on the SMD scale.

### 3.2 Comparisons of heterogeneity across meta-analysis types

We fitted hierarchical models that performed random-effects meta-analysis for each continuous-outcome meta-analysis in the data set, on the SMD scale. After adjusting for meta-analysis characteristics as covariates, a hierarchical model assuming a log-*t* distribution with five degrees of freedom led to a DIC value of 19,562, compared with 29,565 for the inverse-gamma model and 19,582 for the log-normal model for *τ*^2^. Thus, the log-*t* regression model for *τ*^2^ appears to be the better choice. The inverse-gamma model seems a poor fit. In this section, we focus on results from fitting the log-*t* model to investigate the influence of meta-analysis characteristics on the extent of heterogeneity in a meta-analysis. To compare levels of between-study heterogeneity across different meta-analysis types, we report ratios of heterogeneity variances *τ*^2^, together with their respective 95% credible intervals (CIs) (Table 2). Each outcome type is reported in contrast to the largest group of general health–related outcomes, and we report each type of intervention comparison in contrast to the largest group evaluating a nonpharmacologic intervention. As a reference category for medical areas, we choose cardiovascular disease, for which the mean estimate of *τ*^2^ was central across medical areas.

Heterogeneity is on average lowest in meta-analyses assessing an obstetric outcome, with evidence of a difference compared with the largest group of meta-analyses comparing general health–related outcomes; the estimated ratio of variances is 0.39 (95% CI: 0.21, 0.69). We find that heterogeneity is higher in meta-analyses examining resource use or hospital stay/processes and internal and external structure-related outcomes than those assessing general health–related outcomes. Similarly, heterogeneity appears higher in meta-analyses with various subjectively measured outcomes including consumption, satisfaction with care, composite end point (including at most one mortality/morbidity end point), and surgical or device-related success/failure, compared with the reference group of meta-analyses assessing general health–related outcomes.

About the types of intervention comparison, studies within meta-analyses evaluating a nonpharmacologic intervention are on average most heterogeneous. We find that heterogeneity is lowest in meta-analyses comparing two pharmacologic interventions.

The estimated ratios of between-study variances in Table 2 suggest that heterogeneity is substantially lower in meta-analyses related to respiratory diseases than in other medical areas. In this data set, heterogeneity is highest in meta-analyses related to cancer; however, only 24 meta-analyses (0.4%) were related to cancer, so we regard this finding with caution.

### 3.3 Predictive distributions for heterogeneity in future meta-analyses

Initially, we report a predictive distribution for a future meta-analysis for a general setting. This was obtained from a Bayesian hierarchical model fitted to all meta-analyses in the data set, including no meta-analysis characteristics as covariates. The fitted distribution for log(*τ*^2^) is *t*(−3.44,2.59^2^,5), which has a median of 0.03 and 95% range of 0.0002–5.16 on the untransformed scale.

Table 3 summarizes a set of predictive *t* distributions for log($\tau_{new}^{2}$), across settings, defined by type of outcome and intervention comparison type for medical areas other than respiratory diseases and cancer, together with summary statistics for $\tau_{new}^{2}$ on the untransformed scale. Sets of predictive distributions for $\tau_{new}^{2}$ in meta-analyses for medical areas of cancer and respiratory diseases are available in the Appendix at www.jclinepi.com (Section A.3). Although the inverse-gamma distribution does not provide the best fit for underlying values of between-study variance in a meta-analysis, we provide predictive inverse-gamma distributions for $\tau_{new}^{2}$ in the Appendix at www.jclinepi.com (Section A.3). These distributions would facilitate Bayesian random-effects meta-analysis with a conjugate prior for the between-study heterogeneity variance. In Bayesian analysis, use of a conjugate prior is sometimes preferred because the resulting posterior distribution is of the same known form as the prior.

**Table 3:** Predictive distributions for log(*τ*^2^) in future meta-analyses related to medical areas other than cancer and respiratory diseases, together with summary statistics for *τ*^2^ on the untransformed scale

Outcome type	Pharmacologic vs. placebo/control	Pharmacologic vs. pharmacologic	Nonpharmacologic (any)
Obstetric outcome	*t*(−4.13,2.34^2^,5); median = 0.016; 95% range = 0.0002–1.86; *N* = 50	*t*(–4.40,2.31^2^,5); median = 0.012; 95% range = 0.0001–1.16; *N* = 46	*t*(–3.99,2.11^2^,5); median = 0.019; 95% range = 0.0003–1.07; *N* = 69
Resource use and hospital stay/process	*t*(−2.55,2.73^2^,5); median = 0.078; 95% range = 0.0004–21.3; *N* = 78	*t*(−2.83,−2.70^2^,5); median = 0.061; 95% range = 0.0003–11.9; *N* = 48	*t*(−2.41,2.57^2^,5); median = 0.089; 95% range = 0.0005–13.3; *N* = 243
Internal and external structure-related outcome	*t*(−2.43,2.50^2^,5); median = 0.086; 95% range = 0.0007–12.9; *N* = 110	*t*(−2.70,2.46^2^,5); median = 0.070; 95% range = 0.0004–8.32; *N* = 17	*t*(−2.29,2.32^2^,5); median = 0.105; 95% range = 0.0009–10.6; *N* = 45
General physical health and adverse event and pain and quality of life/functioning	*t*(−3.16, 2.50^2^,5); median = 0.040; 95% range = 0.0003–7.02; *N* = 631	*t*(−3.44,2.44^2^,5); median = 0.032; 95% range = 0.0002–4.28; *N* = 212	*t*(−3.02,2.27^2^,5); median = 0.050; 95% range = 0.0006–4.00; *N* = 878
Signs/symptoms reflecting continuation/end of condition and infection/onset of new acute/chronic disease	*t*(−3.00,2.50^2^,5); median = 0.048; 95% range = 0.0004–7.56; *N* = 367	*t*(−3.27,2.47^2^,5); median = 0.038; 95% range = 0.0003–5.69; *N* = 133	*t*(−2.86,2.33^2^,5); median = 0.060; 95% range = 0.0006–5.49; *N* = 428
Mental health outcome	*t*(−2.99,2.16^2^,5); median = 0.049; 95% range = 0.0007–4.70; *N* = 174	*t*(−3.27,2.14^2^,5); median = 0.039; 95% range = 0.0005–3.02; *N* = 75	*t*(−3.85,1.93^2^,5); median = 0.058; 95% range = 0.001–2.58; *N* = 280
Biological marker	*t*(−3.41,2.83^2^,5); median = 0.033; 95% range = 0.0001–10.2; *N* = 401	*t*(−3.68,2.78^2^,5); median = 0.027; 95% range = 0.00001–4.95; *N* = 165	*t*(−3.27,2.66^2^,5); median = 0.037; 95% range = 0.0002–7.33; *N* = 417
Various subjectively measured outcomes	*t*(−2.76,2.58^2^,5); median = 0.063; 95% range = 0.0003–12.0; *N* = 61	*t*(−3.03,2.59^2^,5); median = 0.049; 95% range = 0.0002–8.11; *N* = 39	*t*(−2.62,2.41^2^,5); median = 0.074; 95% range = 0.0007–9.06; *N* = 156

*N* denotes the number of meta-analyses of each type in the CDSR data set.

**Fig. 1:**
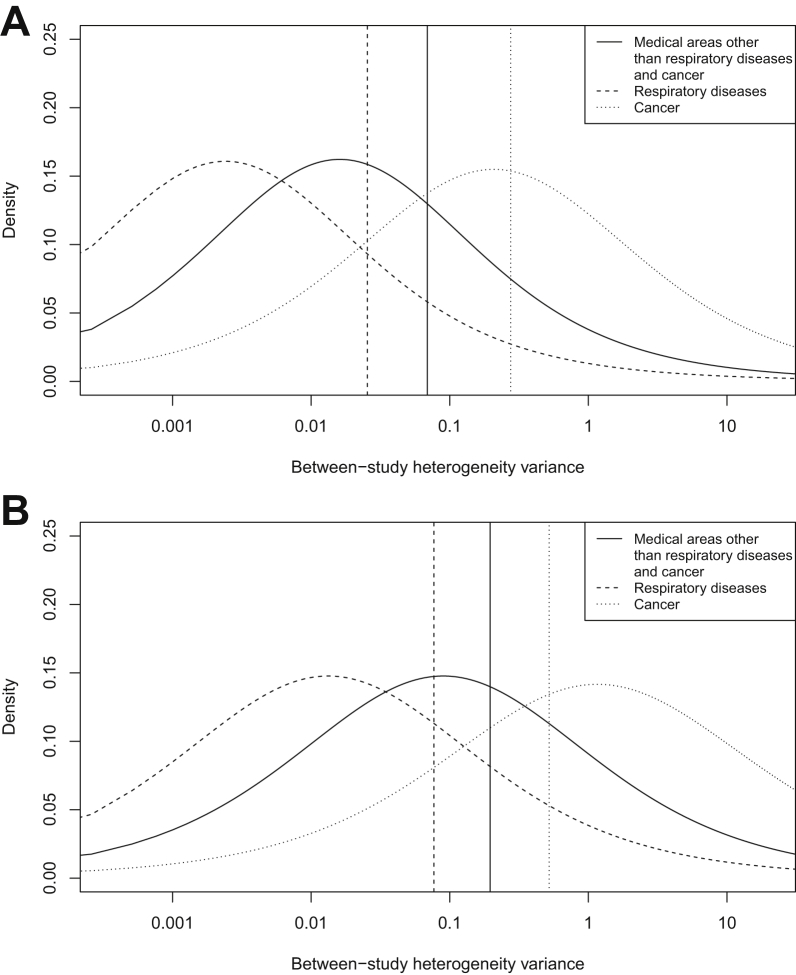
Examples of predictive *t* distributions for the between-study heterogeneity variance (plotted on the log scale). A vertical line highlights the probability of the variance being greater than 1. (A) Pharmacologic vs. placebo/control meta-analyses measuring an obstetric outcome. (B) Nonpharmacologic meta-analyses measuring resource use.

The discrepancies among these fitted distributions reflect the comparisons of between-study variances in Table 2. Fig. 1 illustrates the predictive *t* distributions for between-study heterogeneity in two example settings. For a pharmacologic vs. placebo/control meta-analysis measuring an obstetric outcome, the predictive distribution gives little support to values above 1, whereas the predictive distribution for a nonpharmacologic meta-analysis measuring resource use gives moderate support to values of *τ*^2^ up to 10. Additional density plots representing predictive *t* distributions for between-study heterogeneity in a variety of settings are displayed in the Appendix at www.jclinepi.com (Section A.3).

### 3.4 Application to example meta-analyses

**Fig. 2:**
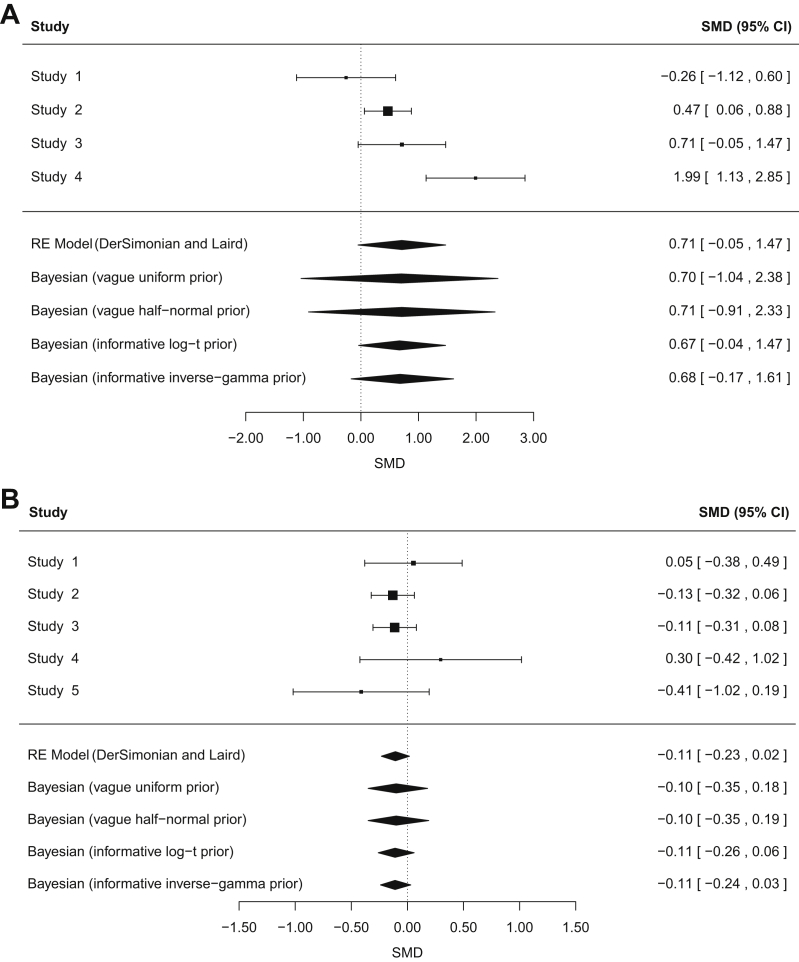
Conventional and Bayesian random-effects meta-analyses combining standardized mean differences (SMDs); 95% confidence intervals (CIs) are shown for each study. (A) Example 1: four studies comparing exercise vs. control (no exercise or placebo exercise) with respect to depression in adults with chronic kidney disease. (B) Example 2: five studies to compare budesonide at different doses for chronic asthma.

To demonstrate the use of an informative prior for the between-study variance *τ*^2^ in a continuous-outcome meta-analysis, we reanalyzed data from two published meta-analyses. Both example meta-analyses represent the typical situation in which there are only a small number of studies in the meta-analysis, and Bayesian estimation is particularly beneficial. The first example meta-analysis consists of just four studies to compare exercise vs. control (no exercise or placebo exercise) with respect to depression in adults with chronic kidney disease (Fig. 2A) [12]. In a conventional random-effects meta-analysis, the heterogeneity is moderately high but imprecisely estimated [*τ*^2^ = 0.47 (95% CI: 0.10, 12.0), *I*^2^ = 79%]. The confidence interval for the conventional estimate of *τ*^2^ was obtained iteratively via the Q-profile method [13].

Results for performing Bayesian random-effects meta-analysis with noninformative priors for heterogeneity are provided in Table 4. As a noninformative prior for the between-study standard deviation *τ*, we used a uniform (0,5) prior, as recommended by Spiegelhalter et al. [11]. We also considered a positive half normal (0,10) distribution for *τ*, which has been used as a prior in earlier applications to meta-analysis [14]. In each Bayesian meta-analysis with a noninformative prior for heterogeneity, the between-study variance is clearly estimated subject to substantial uncertainty, and this is reflected by the wide intervals for the summary intervention effect. This meta-analysis compares a nonpharmacologic intervention against a control in terms of a mental health outcome. A Bayesian meta-analysis implementing an informative log *t*(−3.85,1.93^2^,5) prior for *τ*^2^ leads to a reduced estimate for the between-study heterogeneity of 0.19 (95% CI: 0.001, 2.40). This approach incorporates our beliefs about the likely extent of heterogeneity in this setting, and we therefore consider these results more credible than those obtained using alternative approaches.

**Table 4:** Results from reanalyzing study data from published meta-analyses using conventional and Bayesian approaches to random-effects meta-analysis

Analysis	Summary SMD (95% CI)	Estimated *τ*^2^ (95% CI)
Any exercise vs. control (no exercise/placebo exercise). Outcome: depression		
Conventional random-effects meta-analysis (DerSimonian and Laird estimation)	0.71 (−0.05, 1.47)^a^	0.47 (0.10, 12.0)^a^
Bayesian random-effects meta-analysis with a noninformative uniform (0,5) prior on *τ*	0.70 (−1.04, 2.38)^b^	1.31 (0.09, 15.3)^b^
Bayesian random-effects meta-analysis with a noninformative half normal (0,10) prior on *τ*	0.71 (−0.91, 2.33)^b^	1.15 (0.09, 12.9)^b^
Bayesian random-effects meta-analysis with an informative *t*(−3.85,1.93^2^,5)^c^ prior on log(*τ*^2^)	0.67 (−0.04, 1.47)^b^	0.19 (0.001, 2.40)^b^
Bayesian random-effects meta-analysis with an informative IG (0.46,0.01)^c^ prior on *τ*^2^	0.68 (−0.17, 1.61)^b^	0.29 (0.01, 3.50)^b^
Higher dose budesonide vs. lower dose. Outcome: FEV_1_ measurement		
Conventional random-effects meta-analysis (DerSimonian and Laird estimation)	−0.11 (−0.23, 0.02)^a^	0 (0, 0.45)^a^
Bayesian random-effects meta-analysis with a noninformative uniform (0,5) prior on *τ*	−0.10 (−0.35, 0.18)^b^	1.1 (<0.001, 0.48)^b^
Bayesian random-effects meta-analysis with a noninformative half normal (0,10) prior on *τ*	−0.10 (−0.35, 0.19)^b^	0.01 (<0.001, 0.49)^b^
Bayesian random-effects meta-analysis with an informative *t*(−5.18,2.47^2^,5)^d^ prior on log(*τ*^2^)	−0.11 (−0.26, 0.06)^b^	0.002 (<0.001, 0.06)^b^
Bayesian random-effects meta-analysis with an informative IG (0.94,0.00005)^d^ prior on *τ*^2^	−0.11 (−0.24, 0.03)^b^	<0.001 (<0.001, 0.01)^b^

a 95% confidence interval. For *τ*^2^, this interval is obtained iteratively via the Q-profile method [13].

b Posterior medians and 95% credible intervals are reported.

c Predictive distribution for a nonpharmacologic meta-analysis for a urogenital condition with respect to mental health.

d Predictive distribution for a pharmacologic vs. pharmacologic meta-analysis for respiratory disease with respect to a sign reflecting continuation of condition.

Also presented in Table 4 are results from Bayesian meta-analysis using the corresponding inverse-gamma distribution as an informative prior for *τ*^2^. The simple code for fitting each of the Bayesian models using informative priors for the between-study variance is available in the Appendix at www.jclinepi.com (Section A.4). Central estimates for the summary SMD are similar, irrespective of the form of the prior distribution for heterogeneity. We note that central estimates for the between-study variance are also quite comparable across results from performing Bayesian meta-analysis with log-*t* or inverse-gamma prior distributions. However, there are noticeable discrepancies between the 95% intervals for both the combined SMD and *τ*^2^.

As a contrasting example, we also reanalyzed data from a published meta-analysis consisting of just five studies to compare budesonide at different doses for chronic asthma (Fig. 2B) [15]. In a conventional random-effects meta-analysis, the heterogeneity is low but again imprecisely estimated [*τ*^2^ = 0 (95% CI: 0, 0.45), *I*^2^ = 0%]. Bayesian meta-analysis using an informative log *t*(−5.18,2.47^2^,5) prior for *τ*^2^ leads to a slightly increased estimate for the between-study heterogeneity of 0.002 (95% CI: <0.001, 0.06). Although the central estimate for *τ*^2^ is only a little higher than in the conventional meta-analysis, this approach leads to a wider interval for the summary SMD because it allows appropriately for the uncertainty in between-study heterogeneity.

For this example in which the conventional heterogeneity estimate is low, central estimates and intervals for the summary SMD and the between-study variance show strong similarity between Bayesian meta-analyses using a log-*t* or inverse-gamma prior distribution for the between-study heterogeneity variance.

## 4 Discussion

In this work, we have analyzed data from 6,492 continuous-outcome meta-analyses to describe predictors of heterogeneity and construct informative prior distributions for the between-study variance. We have demonstrated how these priors can be implemented in a Bayesian meta-analysis and given examples in which the precision of heterogeneity is improved with their use.

The results of the present study are consistent with those of the earlier work published on binary outcome meta-analyses [5]. This is to be expected because under often-plausible assumptions, there is a close relationship between the log odds ratio and the SMD [16]. Taken together, there is strong evidence to suggest that the magnitude of heterogeneity in a meta-analysis is substantially influenced by meta-analysis characteristics. Notably, levels of heterogeneity were highest among meta-analyses with subjective outcomes and meta-analyses comparing nonpharmacologic interventions. The current research adds to the existing literature by providing informative log-*t* and inverse-gamma prior distributions for *τ*^2^ in continuous-outcome meta-analyses. The inverse-gamma distributions would facilitate Bayesian meta-analysis with a conjugate prior for the between-study variance. In two example meta-analyses, Bayesian meta-analysis with an informative prior for heterogeneity led to more precise estimates for heterogeneity and results were similar, regardless of the distribution of the informative prior.

An important limitation lies in the fact that there are insufficient data for meta-analyses with certain characteristics. Given the rather extreme levels of heterogeneity observed and the low frequencies of meta-analyses specializing in cancer and respiratory diseases for many settings, we would be cautious about using our informative prior distributions in future meta-analyses related to cancer or respiratory diseases. A well-established problem in conducting Bayesian meta-analysis is the sensitivity of results to priors for variance components [17]. Where the number of past meta-analyses informing the chosen prior is small, we recommend assessing the sensitivity of meta-analysis results to the choice of prior distribution for heterogeneity, using a range of different prior distributions. In addition to using a prior from the Appendix at www.jclinepi.com, an analyst could implement the prior for a general setting. In cases where no relevant data-based prior is available, researchers could use elicited opinion from experts to construct an informative prior for heterogeneity among studies in the meta-analysis.

An issue that has not been addressed in this article is that results for the influence of medical area are highly prone to confounding. All Cochrane reviews in the CDSR have been prepared by authors, under the supervision of a Cochrane Review Group (CRG) in the Cochrane Collaboration. Because CRGs focus on a specific topic area, differences observed between disease areas may be caused by CRG editorial policies. Our results show extremely high heterogeneity among meta-analyses for cancer in comparison with other medical areas. Further examination of these meta-analyses revealed that this high estimate could be due to meta-analyses included in a single Cochrane review [18] with low-quality studies. These meta-analyses show extremely high moment estimators for between-study heterogeneity. Removal of these meta-analyses would be an option, but we expect such examples to be present in other parts of our data set and do not consider such selective omission of data to be appropriate. In any case, we acknowledge the inclusion of such studies in our analyses as a weakness of our work and advise that our priors be used with caution.

A limitation of this work is that the reported informative prior distributions for *τ*^2^ are restricted to use in meta-analyses performed on the SMD scale. As a simple solution, we could transform the between-study heterogeneity variance based on the SMD scale to that based on the MD scale by multiplying by a “typical” within-study standard deviation. However, it is difficult to obtain a good estimate for a “typical” standard deviation of outcome among participants in a study. An alternative to mean difference measures would be to use relative effects by computing a ratio of mean (RoM) values. Although the RoM may be desirable for ease of interpretation and statistical properties [19], we have used the SMD scale throughout our analyses. The RoM is restricted to use in studies in which the means on the two treatment arms have the same sign because we compute the RoM on the natural logarithm scale for mathematical convenience. What are now needed are informative priors for heterogeneity in meta-analyses performed on alternative scales. Higgins and Thompson [20] proposed *I*^2^ as a statistic to quantify the degree of inconsistency among results of included studies in a meta-analysis. This commonly reported measure of inconsistency directly relates to the between-study variance and has the same interpretation regardless of the scale on which meta-analysis is performed. Although it is convenient to assign a prior to *τ*^2^, where possible, because this parameter is used in the analysis, we plan to construct informative prior distributions for *I*^2^ for use in future meta-analyses using different scales. Empirical evidence on *I*^2^ would provide useful information about the degree to which we would expect inconsistency across studies to reduce, on average, if meta-analysis was performed on a different scale or using a different type of outcome data.

In summary, between-study heterogeneity was found to be strongly influenced by the type of outcome measured in the meta-analysis. Informative priors for heterogeneity would be useful in meta-analyses including few studies. Taking into account the important influences of meta-analysis characteristics on heterogeneity, implementing an informative prior for the between-study variance in a new meta-analysis would be beneficial in many settings.
