# Supplementary material for: Predictive distributions were developed for the extent of heterogeneity in meta-analyses of continuous outcome data
Source: J Clin Epidemiol. 2015 Jan;68(1):52–60. doi: 10.1016/j.jclinepi.2014.08.012 (PMC4270451; doi:10.1016/j.jclinepi.2014.08.012)
Supplement: Supplementary Material [file mmc1.pdf]

Appendix (Supplementary material)

A.1 Statistical analysis

A.1.1 Standardized mean difference

Results of each study include the number of participants in each of the two intervention arms, their mean response and the standard deviation of their mean responses (Table A.1).

Study *i*	Mean response	Standard deviation	No. of participants
Control	m_i^C	sd_i^C	n_i^C
Intervention	m_i^T	sd_i^T	n_i^T

Table A.1: Continuous outcome data

Denote the pooled standard deviation across the two intervention groups

$$s_i = \sqrt{\frac{(n_i^C - 1)(sd_i^C)^2 + (n_i^T - 1)(sd_i^T)^2}{N_i - 2}} ,$$

where $N_i = n_i^C + n_i^T$.

The mean difference is given by

$$MD_i = m_i^T - m_i^C .$$

The standardized mean difference (SMD) is

$$SMD_i = \frac{MD_i}{s_i}\left(1 - \frac{3}{4N_i - 9}\right),$$

with standard error

$$SE(SMD_i) = \sqrt{\frac{N_i}{n_i^C n_i^T} + \frac{SMD_i^2}{2(N_i - 3.94)}} .$$

A.1.2 Fitted hierarchical model

We describe the statistical models fitted to study data from all meta-analyses in the data set. Within each meta-analysis with outcome *j* corresponding to pair-wise comparison *k*, a random-effects model with normal within-study likelihoods was fitted to continuous outcome data y_{kji} from each study *i*, on the standardized mean difference (SMD) scale:

$$y_{kji} \sim N(\theta_{kji}, \sigma^2_{kji})$$
$$\theta_{kji} \sim N(\mu_{kj}, \tau^2_{kj}).$$

In the defined model, θ_{kji} denotes the underlying intervention effect for the *i*-th study within outcome *j* within pair-wise comparison *k*, μ_{kj} is the combined intervention effect for a meta-analysis with outcome *j* corresponding to pair-wise comparison *k* and τ^2_{kj} is the corresponding between-study heterogeneity variance.

Across meta-analyses, a hierarchical regression model was fitted to log(τ^2_{kj}), assuming a *t*-distribution with 5 degrees of freedom for the residual variation. In the model below, $x_{1kj},...,x_{8kj}$ are indicators for the type of outcome examined by meta-analysis *j* within pair-wise intervention comparison *k*. Fixed effects $\beta_1,...,\beta_7$ estimate average differences among eight outcome types, whilst the error terms $e_1,...,e_8$ allow for residual variation across meta-analyses with separate variances $\phi^2_1,...,\phi^2_8$ assumed for each outcome type. Similarly z_{1k}, z_{2k}, z_{3k} are binary indicators for the type of intervention comparison under pair-wise comparison *k*. Fixed effects γ_1 and γ_2 estimate average differences among three intervention comparison types, while the random effects u_1,u_2,u_3 allow for variability across pair-wise comparisons, with separate variances $\kappa^2_1,\kappa^2_2,\kappa^2_3$ assumed for each intervention comparison type. Covariates $a_{1kj},...,a_{10kj}$ are indicators for medical areas, with fixed effects $\delta_1,...,\delta_{10}$ estimating average differences among medical specialities.

The log-*t* model fitted to τ^2_{kj} was:

$$\log(\tau^2_{kj}) = \alpha_k + \beta_1 x_{1kj} + ... + \beta_7 x_{7kj} + \delta_1 a_{1kj} + ... + \delta_{10} a_{10kj} + \sum_{l=1}^{8} e_{lkj} x_{lkj}$$

$$\text{and } \alpha_k = \alpha + \gamma_1 z_{1k} + \gamma_2 z_{2k} + \sum_{m=1}^{3} u_{mk} z_{mk},$$

where $e_{lkj} \sim t(0, \phi^2_l, 5)$ and $u_{mk} \sim t(0, \kappa^2_m, 5)$, for *l*=1,2,...,8 and *m*=1,2,3.

For each setting defined by outcome type, type of intervention comparison and medical area, we obtained a predictive distribution for heterogeneity in a new meta-analysis in that setting, within the full Bayesian model:

$$\log(\tau^2_{new}) \sim t(\alpha_{new} + \beta x_{kj} + \delta a_{kj}, \phi^2_l, 5)$$
$$\alpha_{new} \sim t(\alpha + \gamma z_k, \kappa^2_m, 5).$$

We used WinBUGS to obtain a sample from the posterior distribution of log (τ^2_{new}) after convergence. To approximate the predictive distribution for heterogeneity, we used the R function *fitdistr* in the library *MASS* to fit a *t* distribution with 5 degrees of freedom to this sample of values for log (τ^2_{new}). This process provided parametric distributions approximating the predictive distributions, and these distributions were easily summarized and reported for use as priors in new meta-analyses.

A.1.3 WinBUGS code to fit a simple hierarchical model

The following code fits a simple Bayesian hierarchical model to full study-level data, estimating variation in heterogeneity without adjustment for meta-analysis characteristics as covariates.

```
model {
	for (i in 1:N) {
			y[i]~dnorm(theta[i],prec.y[i])
			prec.y[i]<-1/v[i]
			theta[i] ~ dnorm(mu[ma[i]],invtausq[ma[i]])   # Random effects model within meta-analyses
			}
	for (m in 1:M) {
			mu[m] ~ dnorm(0,0.1)
			invtausq[m] <- 1 / tausq[m]
			tausq[m] <- exp(logtausq[m])
			logtausq[m] ~ dt(amongma.mu[comparison[m]],amongma.prec,5)
			}
	for (j in 1:C) {
			amongma.mu[j] ~ dt(mu.all,amongcomp.prec,5)
			}

# Priors for unknown parameters

	mu.all ~ dnorm(0,0.1)      # vague prior for location parameter
	amongma.prec ~ dgamma(0.1,0.1)  # Inverse-gamma prior assumed for scale parameter of t distribution
	amongcomp.prec ~ dgamma(0.1,0.1)

# Obtain a predictive distribution for tausq expected in a new meta-analysis

	logtausq.new ~ dt(amongma.mu.new,amongma.prec,5)
	amongma.mu.new ~ dt(mu.all,amongcomp.prec,5)
	tausq.new <- exp(logtausq.new)
 }
```

A.2 Method-of-moments estimates for between-study variance τ^2

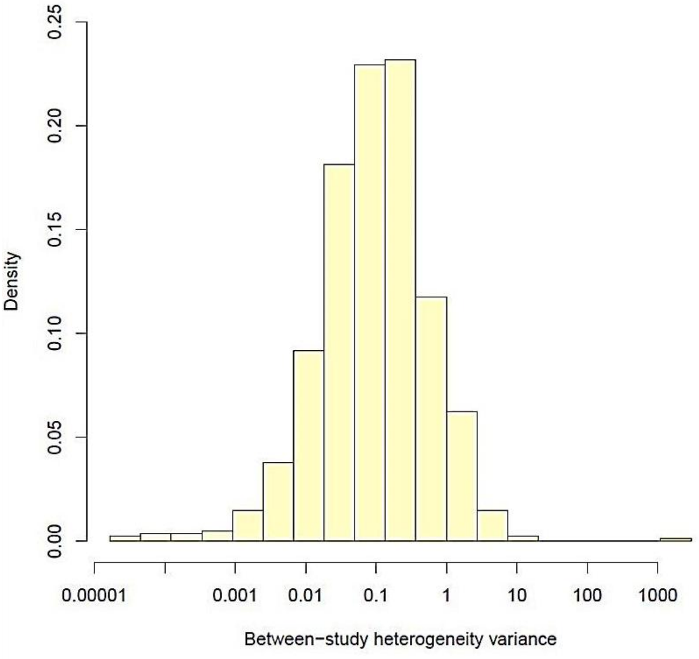

(a) Data published on the SMD scale from 1360 meta-analyses.

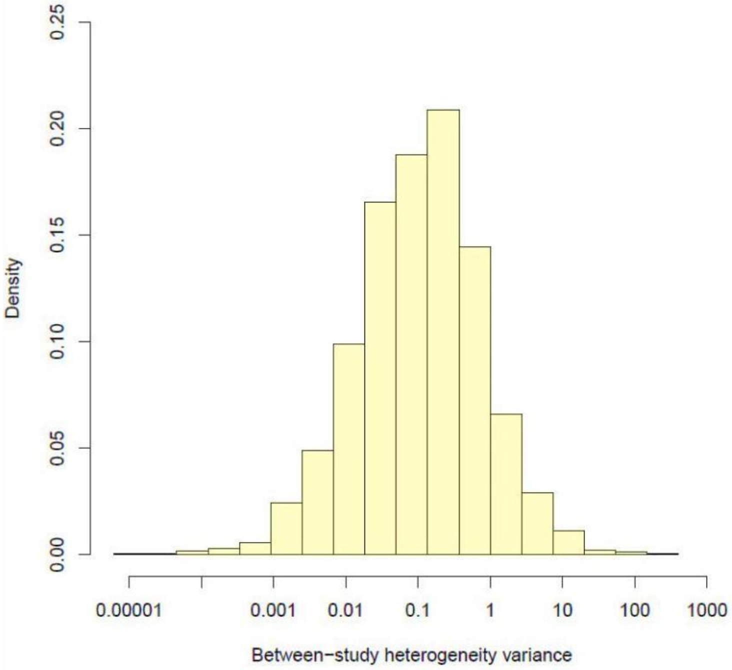

(b) Data published on the mean difference scale from 5132 meta-analyses.

Figure A.2: Distribution of non-zero estimates for the between-study heterogeneity variance τ^2, plotted on the log scale.

A.3 Predictive distributions for heterogeneity in future meta-analyses

We summarize a set of predictive *t*-distributions for $\log(\tau^2)$ in future meta-analyses, across settings defined by type of outcome and type of intervention comparison, together with summary statistics for τ^2 on the untransformed scale, in meta-analyses related to respiratory diseases (Table A.3.1) and cancer (Table A.3.2). Figure A.3 illustrates the predictive *t* distributions for between-study heterogeneity in a variety of settings.

	**Pharmacological Vs. Placebo/ Control**	**Pharmacological Vs. Pharmacological**	**Non-Pharmacological (Any)**
**Obstetric outcome**	$t(-6.03,2.36^2,5)$<br>Median = 0.002; 95% range = 0.00002 to 0.27<br>N=0	$t(-6.31,2.31^2,5)$<br>Median = 0.002; 95% range = 0.00002 to 0.17<br>N=0	$t(-5.89,2.21^2,5)$<br>Median = 0.003; 95% range = 0.00004 to 0.16<br>N=0
**Resource use & hospital stay/process**	$t(-4.46,2.74^2,5)$<br>Median = 0.012; 95% range = 0.00005 to 3.20<br>N=24	$t(-4.73,2.70^2,5)$<br>Median = 0.009; 95% range = 0.00004 to 1.83<br>N=7	$t(-4.32,2.57^2,5)$<br>Median = 0.014; 95% range = 0.00008 to 2.00<br>N=48
**Internal & External structure related outcome**	$t(-4.33,2.51^2,5)$<br>Median = 0.013; 95% range = 0.0001 to 1.99<br>N=1	$t(-4.61,2.46^2,5)$<br>Median = 0.010; 95% range = 0.0006 to 1.32<br>N=0	$t(4.19,2.33^2,5)$<br>Median = 0.016; 95% range = 0.0001 to 1.59<br>N=2
**General physical health & Adverse event & Pain & Quality of life/functioning**	$t(-5.07,2.51^2,5)$<br>Median = 0.006; 95% range = 0.00005 to 1.00<br>N=261	$t(-5.34,2.45^2,5)$<br>Median = 0.005; 95% range = 0.00004 to 0.69<br>N=266	$t(-4.93,2.28^2,5)$<br>Median = 0.007; 95% range = 0.0001 to 0.64<br>N=234
**Signs/symptoms reflecting continuation/end of condition & Infection/onset of new acute/chronic disease**	$t(-4.90,2.50^2,5)$<br>Median = 0.007; 95% range = 0.00005 to 1.15<br>N=160	$t(-5.18,2.47^2,5)$<br>Median = 0.006; 95% range = 0.00004 to 0.83<br>N=161	$t(-4.76,2.33^2,5)$<br>Median = 0.009; 95% range = 0.00008 to 0.81<br>N=50
**Mental health outcome**	$t(-4.90,2.17^2,5)$<br>Median = 0.007; 95% range = 0.0001 to 0.71<br>N=0	$t(-5.17,2.14^2,5)$<br>Median = 0.006; 95% range = 0.00008 to 0.43<br>N=0	$t(-4.76,1.94^2,5)$<br>Median = 0.009; 95% range = 0.0002 to 0.37<br>N=15
**Biological-marker**	$t(-5.31,2.83^2,5)$<br>Median = 0.005; 95% range = 0.00002 to 1.53<br>N=26	$t(-5.59,2.78^2,5)$<br>Median = 0.004; 95% range = 0.00001 to 0.76<br>N=15	$t(-5.17,2.66^2,5)$<br>Median = 0.006; 95% range = 0.00003 to 1.10<br>N=23
**Various subjectively measured outcomes**	$t(-4.66,2.59^2,5)$<br>Median = 0.009; 95% range = 0.00005 to 1.91<br>N=34	$t(-4.94,2.59^2,5)$<br>Median = 0.007; 95% range = 0.00004 to 1.24<br>N=19	$t(-4.52,2.41^2,5)$<br>Median = 0.011; 95% range = 0.0001 to 1.34<br>N=9

Table A.3.1: Medical area of respiratory diseases: Predictive *t* distributions for log (τ^2) in future meta-analyses, together with summary statistics for τ^2 on the untransformed scale. N denotes the number of meta-analyses of each type in the CDSR data set.

	**Pharmacological Vs. Placebo/ Control**	**Pharmacological Vs. Pharmacological**	**Non-Pharmacological (Any)**
**Obstetric outcome**	$t(-1.57,2.45^2,5)$ Median = 0.21; 95% range = 0.002 to 26.2 N=0	$t(-1.85,2.41^2,5)$ Median = 0.16; 95% range = 0.001 to 16.1 N=0	$t(-1.43,2.24^2,5)$ Median = 0.24; 95% range = 0.003 to 18.7 N=0
**Resource use & hospital stay/process**	$t(0.01,2.83^2,5)$ Median = 0.96; 95% range = 0.004 to 360 N=2	$t(-0.27,2.79^2,5)$ Median = 0.77; 95% range = 0.003 to 190 N=0	$t(0.15,2.68^2,5)$ Median = 1.11; 95% range = 0.006 to 209 N=6
**Internal & External structure related outcome**	$t(-0.13,2.61^2,5)$ Median = 1.10; 95% range = 0.008 to 206 N=0	$t(-0.14,2.56^2,5)$ Median = 0.87; 95% range = 0.0006 to 120 N=0	$t(0.27,2.45^2,5)$ Median = 1.33; 95% range = 0.0001 to 172 N=0
**General physical health & Adverse event & Pain & Quality of life/functioning**	$t(-0.60,2.61^2,5)$ Median = 0.53; 95% range = 0.003 to 104 N=2	$t(-0.88,2.55^2,5)$ Median = 0.40; 95% range = 0.003 to 66.3 N=0	$t(-0.46,2.40^2,5)$ Median = 0.65; 95% range = 0.005 to 76.2 N=6
**Signs/symptoms reflecting continuation/end of condition & Infection/onset of new acute/chronic disease**	$t(-0.44,2.60^2,5)$ Median = 0.65; 95% range = 0.004 to 120 N=0	$t(-0.71,2.57^2,5)$ Median = 0.52; 95% range = 0.003 to 84.1 N=0	$t(-0.30,2.46^2,5)$ Median = 0.74; 95% range = 0.005 to 91.4 N=1
**Mental health outcome**	$t(-0.43,2.28^2,5)$ Median = 0.63; 95% range = 0.008 to 63.5 N=0	$t(-0.71,2.25^2,5)$ Median = 0.49; 95% range = 0.006 to 43.9 N=0	$t(-0.29,2.08^2,5)$ Median = 0.75; 95% range = 0.01 to 42.6 N=1
**Biological-marker**	$t(-0.85,2.93^2,5)$ Median = 0.44; 95% range = 0.001 to 156 N=2	$t(-1.13,2.87^2,5)$ Median = 0.34; 95% range = 0.001 to 82.5 N=1	$t(-0.71,2.78^2,5)$ Median = 0.46; 95% range = 0.002 to 119 N=3
**Various subjectively measured outcomes**	$t(-0.20,2.68^2,5)$ Median = 0.79; 95% range = 0.004 to 168 N=0	$t(-0.48,2.68^2,5)$ Median = 0.62; 95% range = 0.003 to 126 N=0	$t(-0.06,2.53^2,5)$ Median = 0.90; 95% range = 0.006 to 147 N=0

Table A.3.2: Medical area of cancer: Predictive t distributions for log (τ^2) in future meta-analyses, together with summary statistics for τ^2 on the untransformed scale. N denotes the number of meta-analyses of each type in the CDSR data set.

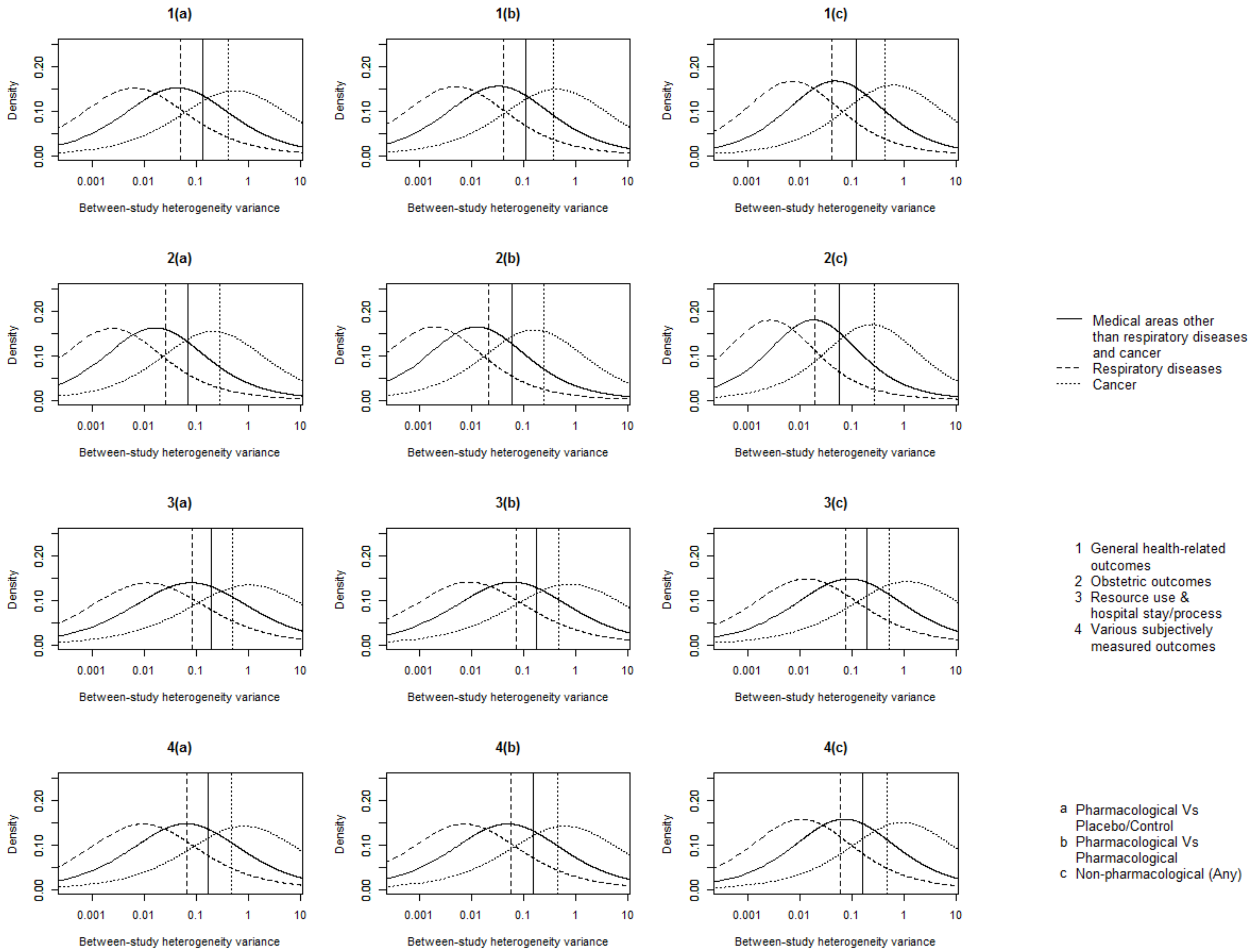

Figure A.3: Examples of predictive *t* distributions for the between-study heterogeneity variance (plotted on the log scale). A vertical line highlights the probability of the variance being greater than 1.

A set of predictive inverse-gamma distributions for τ^2 in future meta-analyses, across settings defined by outcome type and type of intervention comparison, are summarized in Tables A.3.3, A.3.4 and A.3.5, together with summary statistics for τ^2 on the untransformed scale.

	**Pharmacological Vs. Placebo/ Control**	**Pharmacological Vs. Pharmacological**	**Non-Pharmacological (Any)**
**Obstetric outcome**	*IG*(0.11,0.00004)<br>Median = 0.001; 95% range = 0.00004 to 0.09<br>N=0	*IG*(0.36,0.0003)<br>Median = 0.002; 95% range = 0.0001 to 0.31<br>N=0	*IG*(0.42,0.0007)<br>Median = 0.004; 95% range = 0.0003 to 0.58<br>N=0
**Resource use & hospital stay/process**	*IG*(0.31,0.00002)<br>Median = 0.006; 95% range = 0.00001 to 0.65<br>N=24	*IG*(0.39,0.002)<br>Median = 0.012; 95% range = 0.001 to 2.43<br>N=7	*IG*(0.50,0.007)<br>Median = 0.028; 95% range = 0.003 to 4.51<br>N=48
**Internal & External structure related outcome**	*IG*(0.31,0.002)<br>Median = 0.015; 95% range = 0.001 to 3.90<br>N=1	*IG*(0.27,0.000006)<br>Median = 0.004; 95% range = 0.000003 to 24.5<br>N=0	*IG*(0.16,0.007)<br>Median = 0.019; 95% range = 0.0004 to 6.34<br>N=2
**General physical health & Adverse event & Pain & Quality of life/functioning**	*IG*(0.30,0.002)<br>Median = 0.005; 95% range = 0.0006 to 0.50<br>N=261	*IG*(0.37,0.001)<br>Median = 0.008; 95% range = 0.0006 to 0.55<br>N=266	*IG*(0.38,0.002)<br>Median = 0.015; 95% range = 0.0009 to 0.52<br>N=234
**Signs/symptoms reflecting continuation/end of condition & Infection/onset of new acute/chronic disease**	*IG*(0.42,0.0001)<br>Median = 0.006; 95% range = 0.00005 to 1.19<br>N=160	*IG*(0.94,0.00005)<br>Median = 0.00007; 95% range = 0.00002 to 0.11<br>N=161	*IG*(0.29,0.0006)<br>Median = 0.013; 95% range = 0.0003 to 0.48<br>N=50
**Mental health outcome**	*IG*(0.42,0.00002)<br>Median = 0.002; 95% range = 0.000009 to 0.25<br>N=0	*IG*(0.30,0.0009)<br>Median = 0.007; 95% range = 0.0006 to 0.43<br>N=0	*IG*(0.44,0.003)<br>Median = 0.013; 95% range = 0.001 to 0.60<br>N=15
**Biological-marker**	*IG*(0.28,0.00007)<br>Median = 0.004; 95% range = 0.00004 to 1.79<br>N=26	*IG*(0.28,0.0002)<br>Median = 0.002; 95% range = 0.000009 to 0.42<br>N=15	*IG*(0.46,0.003)<br>Median = 0.013; 95% range = 0.001 to 0.55<br>N=23
**Various subjectively measured outcomes**	*IG*(0.62,0.006)<br>Median = 0.018; 95% range = 0.002 to 1.38<br>N=34	*IG*(0.36,0.003)<br>Median = 0.019; 95% range = 0.001 to 5.81<br>N=19	*IG*(0.61.0.009)<br>Median = 0.024; 95% range = 0.003 to 1.61<br>N=9

Table A.3.3: Medical area of respiratory disease: Predictive inverse-gamma distributions for τ^2 in future meta-analyses, together with summary statistics for τ^2 on the untransformed scale. N denotes the number of meta-analyses of each type in the CDSR data set.

	Pharmacological Vs. Placebo/ Control	Pharmacological Vs. Pharmacological	Non-Pharmacological (Any)
**Obstetric outcome**	*IG*(0.29,0.008)<br>Median = 0.073; 95% range = 0.005 to 33.8<br>N=0	*IG*(0.21,0.005)<br>Median = 0.091; 95% range = 0.004 to 125<br>N=0	*IG*(0.20,0.011)<br>Median = 0.193; 95% range = 0.008 to 255<br>N=0
**Resource use & hospital stay/process**	*IG*(0.24,0.007)<br>Median = 0.160; 95% range = 0.004 to 20.2<br>N=2	*IG*(0.26,0.05)<br>Median = 0.636; 95% range = 0.03 to 1105<br>N=0	*IG*(0.27,0.12)<br>Median = 1.487; 95% range = 0.07 to 2464<br>N=6
**Internal & External structure related outcome**	*IG*(0.20,0.04)<br>Median = 0.758; 95% range = 0.03 to 1823<br>N=0	*IG*(0.08,0.0002)<br>Median = 0.121; 95% range = 0.0002 to 779<br>N=0	*IG*(0.14,0.03)<br>Median = 1.015; 95% range = 0.04 to 1528<br>N=0
**General physical health & Adverse event & Pain & Quality of life/functioning**	*IG*(0.33,0.04)<br>Median = 0.310; 95% range = 0.02 to 17.7<br>N=2	*IG*(0.23,0.02)<br>Median = 0.362; 95% range = 0.01 to 275<br>N=0	*IG*(0.25,0.04)<br>Median = 0.566; 95% range = 0.02 to 543<br>N=6
**Signs/symptoms reflecting continuation/end of condition & Infection/onset of new acute/chronic disease**	*IG*(0.20,0.01)<br>Median = 0.256; 95% range = 0.007 to 33.4<br>N=0	*IG*(0.27,0.0006)<br>Median = 0.050; 95% range = 0.0004 to 3.5<br>N=0	*IG*(0.35,0.09)<br>Median = 0.837; 95% range = 0.04 to 35.2<br>N=1
**Mental health outcome**	*IG*(0.17,0.008)<br>Median = 0.096; 95% range = 0.00005 to 8.68<br>N=0	*IG*(0.20,0.02)<br>Median = 0.315; 95% range = 0.014 to 311<br>N=0	*IG*(0.29,0.05)<br>Median = 0.571; 95% range = 0.03 to 496<br>N=1
**Biological-marker**	*IG*(0.28,0.02)<br>Median = 0.230; 95% range = 0.009 to 36.8<br>N=2	*IG*(0.21,0.004)<br>Median = 0.102; 95% range = 0.002 to 11.0<br>N=1	*IG*(0.29,0.05)<br>Median = 0.560; 95% range = 0.03 to 417<br>N=3
**Various subjectively measured outcomes**	*IG*(0.33,0.11)<br>Median = 0.968; 95% range = 0.059 to 806<br>N=0	*IG*(0.21,0.06)<br>Median = 1.056; 95% range = 0.05 to 2104<br>N=0	*IG*(0.32,0.14)<br>Median = 1.204; 95% range = 0.07 to 1073<br>N=0

Table A.3.4: Medical area of cancer: Predictive inverse-gamma distributions for τ^2 in future meta-analyses, together with summary statistics for τ^2 on the untransformed scale. N denotes the number of meta-analyses of each type in the CDSR data set.

	Pharmacological Vs. Placebo/ Control	Pharmacological Vs. Pharmacological	Non-Pharmacological (Any)
**Obstetric outcome**	*IG*(0.05,0.00004)<br>Median = 0.006; 95% range = 0.00006 to 0.41<br>N=50	*IG*(0.38,0.001)<br>Median = 0.006; 95% range = 0.0006 to 1.25<br>N=46	*IG*(0.44,0.003)<br>Median = 0.014; 95% range = 0.001 to 2.47<br>N=69
**Resource use & hospital stay/process**	*IG*(0.25,0.0004)<br>Median = 0.025; 95% range = 0.00002 to 2.92<br>N=78	*IG*(0.47,0.01)<br>Median = 0.046; 95% range = 0.005 to 8.50<br>N=48	*IG*(0.52,0.03)<br>Median = 0.107; 95% range = 0.01 to 20.5<br>N=243
**Internal & External structure related outcome**	*IG*(0.36,0.009)<br>Median = 0.058; 95% range = 0.005 to 12.6<br>N=110	*IG*(0.31,0.00003)<br>Median = 0.017; 95% range = 0.00001 to 105.8<br>N=17	*IG*(0.08,0.0007)<br>Median = 0.071; 95% range = 0.0006 to 17.4<br>N=45
**General physical health & Adverse event & Pain & Quality of life/functioning**	*IG*(0.21,0.0003)<br>Median = 0.019; 95% range = 0.0002 to 2.22<br>N=631	*IG*(0.36,0.004)<br>Median = 0.032; 95% range = 0.002 to 2.01<br>N=212	*IG*(0.42,0.01)<br>Median = 0.064; 95% range = 0.004 to 1.88<br>N=878
**Signs/symptoms reflecting continuation/end of condition & Infection/onset of new acute/chronic disease**	*IG*(0.64,0.003)<br>Median = 0.022; 95% range = 0.0001 to 3.59<br>N=367	*IG*(1.02,0.0002)<br>Median = 0.0003; 95% range = 0.00007 to 0.37<br>N=133	*IG*(0.17,0.0009)<br>Median = 0.053; 95% range = 0.0005 to 2.09<br>N=428
**Mental health outcome**	*IG*(0.53,0.00009)<br>Median = 0.008; 95% range = 0.00005 to 1.03<br>N=174	*IG*(0.29,0.004)<br>Median = 0.029; 95% range = 0.002 to 2.15<br>N=75	*IG*(0.46,0.01)<br>Median = 0.055; 95% range = 0.005 to 2.20<br>N=1
**Biological-marker**	*IG*(0.36,0.0003)<br>Median = 0.019; 95% range = 0.0002 to 6.12<br>N=401	*IG*(0.37,0.00009)<br>Median = 0.009; 95% range = 0.00005 to 1.27<br>N=165	*IG*(0.48,0.01)<br>Median = 0.056; 95% range = 0.005 to 2.81<br>N=417
**Various subjectively measured outcomes**	*IG*(0.63,0.03)<br>Median = 0.071; 95% range = 0.009 to 5.06<br>N=61	*IG*(0.43,0.02)<br>Median = 0.076; 95% range = 0.007 to 26.2<br>N=39	*IG*(0.68,0.04)<br>Median = 0.095; 95% range = 0.02 to 8.07<br>N=156

Table A.3.5: Medical areas other than cancer and respiratory diseases: Predictive inverse-gamma distributions for τ^2 in future meta-analyses, together with summary statistics for τ^2 on the untransformed scale. N denotes the number of meta-analyses of each type in the CDSR data set.

A.4 WinBUGS code for the application to an example meta-analysis

Bayesian meta-analysis of data from studies evaluating the effectiveness of an exercise intervention for preventing depression [12], incorporating an informative prior for the between-study variance τ^2 .

Meta-analysis implementing a log-t prior for the between-study variance

```
model{
        for(i in 1:K){
                MD[i]<-treat_mean[i]-ctrl_mean[i]
                s[i]<-sqrt((((nT[i]-1)*treat_sd[i]*treat_sd[i])
                  +((nC[i]-1)*ctrl_sd[i]*ctrl_sd[i]))/(N[i]-2))
                N[i]<-nC[i]+nT[i]
                y[i]<-(MD[i]/s[i])*(1-(3/(4*N[i]-9)))
                v[i]<-(N[i]/(nC[i]*nT[i]))+((y[i]*y[i])/(2*(N[i]-3.94)))
                prec.y[i]<-1/v[i]
                y[i]~dnorm(theta[i],prec.y[i]) # Normal distribution for observed study-level SMDs
                theta[i]~dnorm(mu,invtausq) # Random-effects meta-analysis model
                    }
                mu~dnorm(0,0.000001) # Vague prior for summary intervention effect (SMD scale)

                #implement an informative prior for log(tau-squared)
                invtausq<-1/tausq
                tausq<-exp(log.tausq)
                prior.prec<-1/(1.93*1.93)
                log.tausq~dt(-3.85, prior.prec,5)
              # t(5df) prior with location parameter -3.85 and scale parameter 1.93
       }
```

Meta-analysis implementing an inverse-gamma prior for the between-study variance

```
model{
        for(i in 1:K){
                MD[i]<-treat_mean[i]-ctrl_mean[i]
                s[i]<-sqrt((((nT[i]-1)*treat_sd[i]*treat_sd[i])
                  +((nC[i]-1)*ctrl_sd[i]*ctrl_sd[i]))/(N[i]-2))
                N[i]<-nC[i]+nT[i]
                y[i]<-(MD[i]/s[i])*(1-(3/(4*N[i]-9)))
                v[i]<-(N[i]/(nC[i]*nT[i]))+((y[i]*y[i])/(2*(N[i]-3.94)))
                prec.y[i]<-1/v[i]
                y[i]~dnorm(theta[i],prec.y[i]) # Normal distribution for observed study-level SMDs
                theta[i]~dnorm(mu,invtausq) # Random-effects meta-analysis model
                    }
                mu~dnorm(0,0.000001) # Vague prior for summary intervention effect (SMD scale)

                #implement an informative prior for tau-squared
                tausq<-1/invtausq
                invtausq~dgamma(0.46, 0.01)
                            # IG prior with shape parameter 0.46 and scale parameter 0.01
  }
```

Depression data for 4 studies:

```
list(K=4,nT=c(11,43,11,14),nC=c(10,53,20,19),treat_mean=c(5,41.4,21.3,19.4),
ctrl_mean=c(6.8,37.2,13.7,11.7),treat_sd=c(5,9.6,11.9,4),ctrl_sd=c(8.2,8.3,9.5,3.6))
```
